# Weight Loss Surgery Utilization in Patients Aged 14–25 With Severe Obesity Among Several Healthcare Institutions in the United States

**DOI:** 10.3389/fped.2018.00251

**Published:** 2018-09-19

**Authors:** Karen J. Campoverde Reyes, Madhusmita Misra, Hang Lee, Fatima Cody Stanford

**Affiliations:** ^1^Neuroendocrine Unit, Massachusetts General Hospital and Harvard Medical School, Boston, MA, United States; ^2^Liver Research Center, Beth Israel Deaconess Medical Center, Boston, MA, United States; ^3^Pediatric Endocrinology, Massachusetts General Hospital and Harvard Medical School, Boston, MA, United States; ^4^MGH Biostatistics Center, Massachusetts General Hospital, Boston, MA, United States; ^5^MGH Weight Center, Massachusetts General Hospital, Boston, MA, United States

**Keywords:** obesity surgery, weight loss, adolescent, youth, bariatric surgery

## Abstract

**Introduction:** Obesity is associated with early co-morbidities and higher mortality. Even though weight loss surgery (WLS) in adolescents with severe obesity reliably achieves safe and lasting improvement in BMI and superior resolution of comorbid diseases, its utilization among young patients in the clinical practice stands unclear.

**Objective:** To show the prevalence of weight loss surgery utilization rates in adolescents and young adults among several healthcare institutions in the United States.

**Method:** WLS in 14–25 years old between 2000 and 2017 was obtained from Washington University, Morehouse Medical, University of Texas, Wake Forest Baptist Medical Center, Beth Israel Deaconess Medical Center, Boston Children's Hospital, Boston Medical Center, and Partners Healthcare using the Shared Health Research Information Network (SHRINE) and Research Patient Data Registry (RPDR) web-based query tools. ICD-9 codes were used for bariatric surgery.

**Results:** Among 2500635 individuals, 18008 (0.7%) had severe obesity. At Partners, 1879 patients had severe obesity, of which 404 (21.5%) underwent WLS, whereas at Washington University, 44 (2.5%) of 1788 the underwent WLS. 13 (2.3%) of the 575 at BIDMC, 43 (1.5%) of the 2969 at BMC, and 37 (0.4%) of 8908 at BCH underwent WLS (*p* < 0.0001 for all).

**Discussion:** Even though WLS has shown to be the most effective treatment to create sustainable changes in metabolic derangements for moderate to severe obesity and its comorbidities, it has been underutilized. Further studies need to be conducted to ensure WLS is utilized for those patients who would achieve the most benefit.

## Introduction

Obesity rates have reached pandemic levels in the United States with prevalence rates of 39.8% in adults and 18.5% in youth (1). Current reports in children and adolescents show a significant increase in severe obesity in children and an upward trend in many subgroups, including adolescents (2, 3).

Severe obesity is defined as a BMI ≥ 99th percentile for age and gender, equivalent to a BMI Z-score of +2.5, which is an adult BMI of 30 kg/m^2^ (4). A high BMI for age, especially in those with severe obesity, is associated with early co-morbidities, such as type 2 diabetes, non-alcoholic fatty liver disease (NAFLD), non-alcoholic steatohepatitis (NASH), dyslipidemia, obstructive sleep apnea (OSA), hypertension (HTN), heart disease, and early mortality. About 4% of children in the US have a BMI ≥99th (5).

Obesity is the most significant threat to the health of younger generations due to its cumulative health impact (4). Current indications for WLS in adolescents include a BMI ≥35 kg/m^2^ or ≥120% of the 95th percentile with clinically significant comorbid conditions such as OSA (apnea-hypopnea index (AHI) >5 events/h), type 2 diabetes, idiopathic intracranial hypertension, NASH, Blount's disease, slipped capital femoral epiphysis, gastroesophageal reflux disease or HTN; or BMI ≥40 kg/m^2^ or ≥140% of the 95th percentile (6). In adolescents with severe obesity, WLS reliably achieves a safe and lasting improvement in BMI and resolution of comorbid diseases superior to other treatment modalities (7–11). Long term remission of comorbidities such as diabetes, dyslipidemia, HTN, NAFLD, and OSA have been reported in adolescents. WLS is recommended as a reasonably safe and effective approach in adolescents and young adults. (4, 9, 12–15). However, dietary supplementation and lifelong vitamin level monitoring are required since inadequate absorption of calcium, vitamin D, iron, vitamin B1, B6, B12, A, and folate has been reported in these patients (4, 6).

Ensuring a multidisciplinary team (MDT) optimizes preoperative selection and education, as well as postoperative outcomes (6, 16). Many academic institutions in the US do not offer this treatment modality. Even though WLS is the most effective treatment to date to create sustainable changes in metabolic derangements for those with moderate to severe obesity and its comorbidities, its utilization among young patients in the clinical practice remains unclear. Our hypothesis is that WLS in adolescents and young adults with severe obesity is highly underutilized, despite its benefits. In our study, we evaluate the prevalence of severe obesity and WLS utilization in individuals between 14 and 25-years-old among several academic healthcare institutions in the United States.

## Methods

The prevalence of obesity, severe obesity, and WLS utilization between 2000 and 2017 in patients between 14 and 25-years-old were obtained using the Scalable Collaborative Infrastructure for a Learning Health System (SCILHS) and the Research Patient Data Registry (RPDR) query web-based tools. The SCILHS covers more than 8 million patients across 10 health systems, enabling a national research network formed on an advanced information technology infrastructure (17). The RPDR is a centralized clinical data registry that gathers clinical information from various hospitals in the Partners Healthcare system (PHS), such as Massachusetts General Hospital (MGH) and Brigham and Women's Hospital (BWH), covering around 4 million patients.

Data on gender, age, race, and WLS utilization were available from 8 healthcare systems: Washington University in St. Louis, Morehouse Medical School, University of Texas-Houston, Wake Forest Baptist Medical Center, Beth Israel Deaconess Medical Center (BIDMC), Boston Children's Hospital (BCH), Boston Medical Center (BMC), and PHS. Individuals with obesity were identified utilizing ICD-9 code for obesity, BMI>30 kg/m^2^, and BMI pediatric ≥ 95th percentile for age. Individuals with severe obesity were identified using the ICD-9 code for severe obesity, morbid obesity, and BMI ≥40. Data for WLS were also obtained using ICD-9 codes:: gastric restrictive procedure, gastric bypass for morbid obesity; short limb Roux-en-Y with small intestine reconstruction to limit absorption, laparoscopic surgical gastric restrictive procedure with gastric bypass, and Roux-en-Y gastroenterostomy. Statistical analyses involved a series of univariate analyses consisting of Fisher's exact test to evaluate the difference in WLS utilization rates between each healthcare institution and PHS. This study was approved by the Partners Healthcare Institutional Review Board. The study contained only de-identified information, and we have followed the principles in accordance with the Declaration of Helsinki.

## Results

### Prevalence of obesity

Our study collected information on 2,500,638 individuals 14–25 years old. As illustrated in Table 1, we ascertained demographics of the total population, of individuals with obesity and with severe obesity, by gender, race and academic institution. For the total population, gender distribution was overall even. Overall, Partners Healthcare had the highest percentage of individuals between 14-25 years old (38.8%), followed by BCH (22.3%). We found that 18008 (0.7%) patients had severe obesity, of whom 61% were female, 39% were male. By healthcare institution, BCH had the highest proportion of individuals with severe obesity.

**Table 1:** Demographics of individuals by gender, race and healthcare institution.

	**Total Population (*n* = 2500638)**	**Individuals with obesity (*n* = 78867)**	**Individuals with severe obesity (*n* = 18008)**
Female	1271087 (50.8%)	44802 (56.8%)	10967 (61.0%)
Male	1228202 (49.1%)	34059 (43.2%)	7031 (39.0%)
**RACE**			
White	1215940 (48.6%)	32344 (41.0%)	7279 (40.4%)
American Indian	4306 (0.2%)	129 (0.2%)	13 (0.1%)
Asian	75255 (3.0%)	2078 (2.6%)	210 (1.2%)
African American	283826 (11.4%)	17656 (22.4%)	4612 (25.6%)
Pacific Islander	674 (0.02%)	18 (0.02%)	0
Other	241630 (9.6%)	14810 (18.8%)	3107 (17.3%)
Unknown	679004 (27.2%)	11832 (15%)	2787 (15.5%)
**HEALTHCARE INSTITUTIONS**			
BIDMC	139000 (5.6%)	3157 (4.0%)	575 (3.2%)
Wake Forest	173500 (6.9%)	8750 (11.1%)	654 (3.6%)
U Texas	207884 (8.3%)	5239 (6.6%)	1115 (6.2%)
Boston Children's Hospital	558159(22.3%)	21347 (27.1%)	8908 (49.5%)
BMC	112505(4.5%)	8670 (11.0%)	2969 (16.5%)
Washington University	333338(13.3%)	6173 (7.8%)	1788 (9.9%)
Partners Healthcare System	970010(38.8%)	24944 (31.6%)	1879 (10.4%)
Morehouse	6242 (0.3%)	587 (0.7%)	120 (0.7%)

### Utilization of WLS

**Figure 1:**
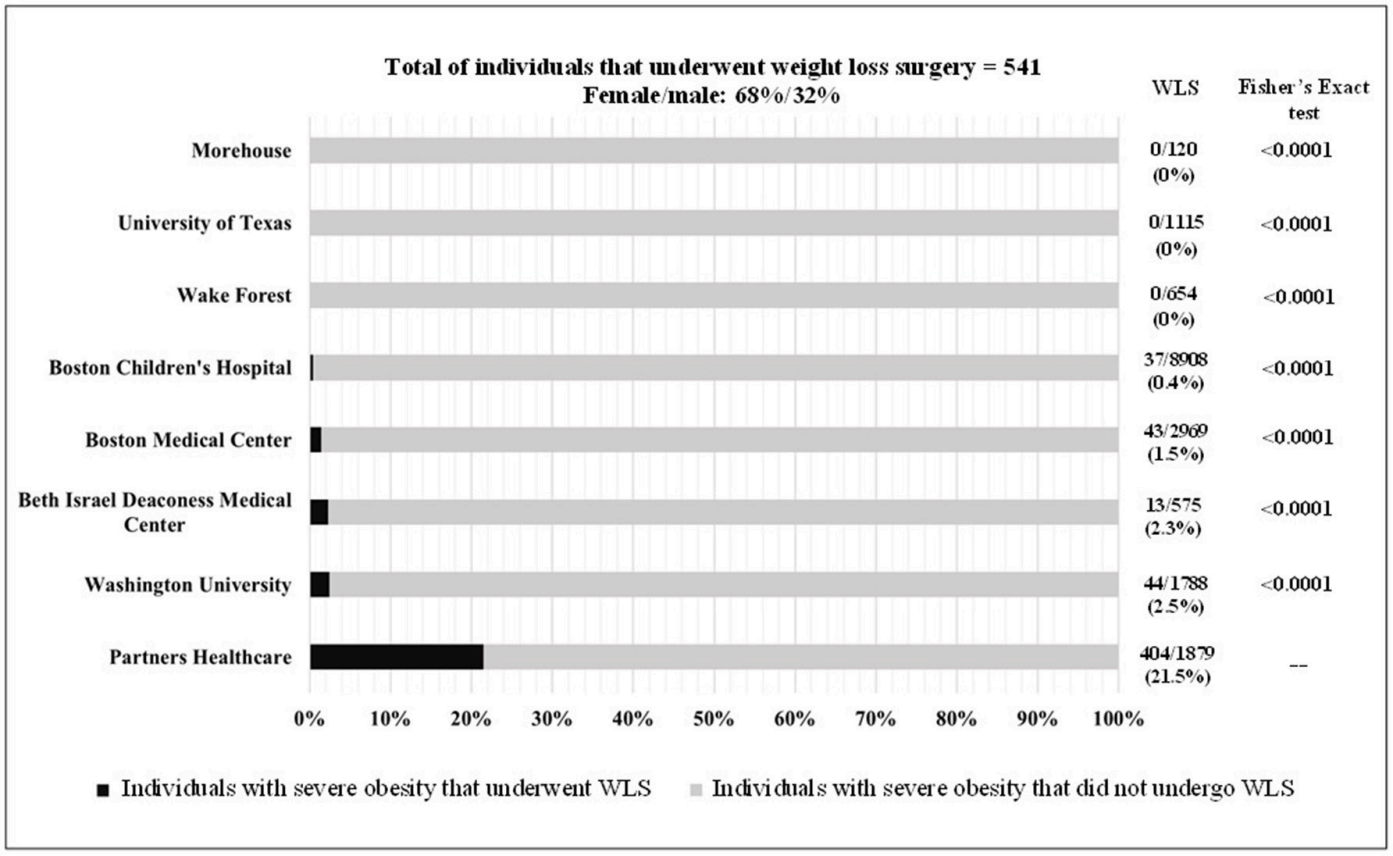
Weight loss surgery utilization in patients 14-25 years old in several healthcare academic institutions in US.

Only 3% of individuals with severe obesity underwent WLS. The gender distribution was 2:1 in favor of women, and utilization was higher in the White population (42.3%), followed by Unknown (24.4%), Other (15.5%), African American (6.5%), and Asian (0.7%). The distribution of WLS by healthcare institution was as follows: at PHS, 404 (21.5%) of 1879 patients with severe obesity underwent WLS. In contrast, 44 (2.5%) of 1788 at Washington University, 13 (2.3%) of the 575 at BIDMC, 43 (1.4%) of the 2969 at BMC, and 37 (0.4%) of 8908 individuals with severe obesity at BCH underwent WLS. As illustrated in Figure 1, we used Fisher's exact test to determine whether each of the other institutions differed from PHS in WLS utilization rates among those with severe obesity. There was a statistically significant difference in WLS utilization rates between each of the other healthcare institutions and PHS (*p* < 0.0001 for all).

## Discussion

Despite evidence that adolescent bariatric surgery is a safe, effective, and appropriate consideration when severe obesity in the adolescent patient does not respond to behavioral changes or medical interventions, our results demonstrate low utilization rates of WLS in adolescent and young adult patients with severe obesity in a nationwide sample of institutions. We compared WLS utilization rates in other academic health care institutions to that within the PHS (based on the obviously higher utilization of WLS in the latter). A vast difference was found among academic institutions, despite some being localized in the same city. Of the institutions evaluated, PHS has the longest history of providing care to patients in a tertiary multidisciplinary weight management program (which includes the availability of weight loss surgery), and this may have influenced its highest percentage of WLS utilization when compared to peer institutions. Patients with severe obesity can only undergo WLS after being carefully selected, and WLS can produce meaningful short term and long term weight loss, improvements in cardiometabolic risk factors, diabetes mellitus, and quality of life (12, 18). To our knowledge, all academic institutions included in our analyses utilize a multidisciplinary weight management program for their patient population.

Numerous factors are believed to impact WLS utilization. These include limited medical education about the physiology of obesity and its treatment, obesity bias with healthcare providers assuming it is the patient's deficient effort to achieve weight loss that results in severe obesity, limited decisional capacity and autonomy in adolescents, a limited ability to manage challenging lifestyle changes, lack of awareness that adolescents and young adults are eligible for WLS, inequities in access to healthcare resources, differences in socioeconomic status (SES), obstacles obtaining treatment authorization from insurance carriers, and limited studies on long-term outcomes (12, 18, 19). The adolescent years are a very vulnerable time of one's life and are characterized by a peak in psychological and social development. Severe obesity may leave a more significant metabolic imprint in adolescents than in adults, for which is imperative to consider WLS as an approach if applicable. In patients with severe obesity, the embracing of a new lifestyle after WLS potentially results in a more energetic, healthy, and sociable young individual, with a better self-esteem and a sense of self-empowerment, however these benefits must always outweigh the risks of micronutrient deficiencies and possibility of more abdominal procedures in the future (7.9%) (4, 12, 13, 20). Limitations of this research included not having access to insurance and SES. Further studies are required to elucidate the reasons of WLS underutilization in an adolescent and young adult population.

## Conclusion

Management of severe obesity is a lifelong challenge and requires a multidisciplinary and individualized approach that includes a combination of lifestyle changes, behavioral therapy, nutrition education, medications, and possibly WLS. WLS is a safe and effective treatment for adolescents and young adults with severe obesity; however, it is widely underutilized. More tertiary multidisciplinary weight management centers are necessary to ensure the beneficial early intervention outcomes of WLS. Inadequate education and awareness, suboptimal support and inadequate tools and access to navigate the decision-making process regarding WLS might influence this outcome. These factors remain unclear and further measures are necessary to ensure that WLS is adequately utilized for those patients who would achieve the most benefit.

## Author contributions

All authors have made substantial contribution to the paper. KC was responsible for acquisition of data, drafting the manuscript and interpreting the data. FS was responsible for responsible for the conception and design of the study and critical review of the paper. HL was responsible of data analyses and interpreting the data. FS and MM were responsible for revising the article critically and adding important intellectual content. All authors have read and approved the final version of the paper.

### Conflict of interest statement

The authors declare that the research was conducted in the absence of any commercial or financial relationships that could be construed as a potential conflict of interest.

## References

[1] Hales CM Carroll MD Fryar CD Ogden CL. Prevalence of obesity among adults and youth: United States, 2015–2016. NCHS Data Brief (2017) 1–8. 29155689

[2] Skinner AC Ravanbakht SN Skelton JA Perrin EM Armstrong SC Prevalence of obesity and severe obesity in US children, 1999–2016. Pediatrics (2018) 141:e20173459 10.1542/peds.2017-3459 PMC6109602 29483202

[3] Hales CM Fryar CD Carroll MD Freedman DS Ogden CL. Trends in obesity and severe obesity prevalence in US youth and adults by sex and age, 2007-2008 to 2015-2016. JAMA (2018) 319:1723–5. 10.1001/jama.2018.3060 29570750 PMC5876828

[4] Durkin N Ashish PD. What is the evidence for paediatric/adolescent bariatric surgery? Curr Obes Rep. (2017) 6:278–5. 10.1007/s13679-017-0277-4 28815416 PMC5585991

[5] Freedman D Mei Z Srinivasan SR Berenson GS Dietz WH. Cardiovascular risk factors and excess adiposity among overweight children: bogalusa heart study. J Pediat. (2007) 150:12–7.e2. 10.1016/j.jpeds.2006.08.042 17188605

[6] Pratt JS Browne A Browne NT Bruzoni M Cohen M Desai A. ASMBS pediatric metabolic and bariatric surgery guidelines, 2018. Surg Obes Relat Dis. (2018) 14:882–901. 10.1016/j.soard.2018.03.019. 30077361 PMC6097871

[7] Campos GM Lanning DA. Bariatric surgery for nonalcoholic fatty liver disease in adolescents with severe obesity. Surg Obes Relat Dis. (2015) 449–450. 10.1016/j.soard.2014.12.015 25817747

[8] Nandagopal R Brown RJ Rother KI. Resolution of type 2 diabetes following bariatric surgery: implications for adults and adolescents. Diab Technol Therapeut. (2010) 12 671–7. 10.1089/dia.2010.0037 20615109 PMC2936261

[9] Maffazioli GD Stanford FC Campoverde Reyes KJ Stanley TL Singhal V Corey KE. Comparing outcomes of two types of bariatric surgery in an adolescent obese population: roux-en-Y gastric bypass vs. Sleeve Gastrectomy. Front Pediatr. (2016) 4:78. 10.3389/fped.2016.00078 27508205 PMC4960456

[10] Inge TH Siegel RM Xanthakos SA. Weight loss maintenance: a hard nut to crack. JAMA Pediatr. (2014) 168:796–7. 10.1001/jamapediatrics.2014.672 25022564

[11] Treadwell JR Sun F Schoelles K. Systematic review and meta-analysis of bariatric surgery for pediatric obesity. Ann Surg. (2008) 248:763–76. 10.1097/SLA.0b013e31818702f4 18948803

[12] Shoar S Mahmoudzadeh H Naderan M Bagheri-Hariri S Wong C Parizi AS. Long-term outcome of bariatric surgery in morbidly obese adolescents: a systematic review and meta-analysis of 950 patients with a minimum of 3 years follow-up. Obes Surg. (2017) 27:3110–7. 10.1007/s11695-017-2738-y 28573535

[13] Khidir N El-Matbouly MA Sargsyan D Al-Kuwari M Bashah M Gagner M. Five-year outcomes of laparoscopic sleeve gastrectomy: a comparison between adults and adolescents. Obes Surg. (2018) 28:2040–5. 10.1007/s11695-018-3139-6 29430596

[14] Buchwald H Estok R Fahrbach K Banel D Jensen MD Pories WJ. Weight and type 2 diabetes after bariatric surgery: systematic review and meta-analysis. Am J Med. (2009) 122:248–56. 10.1016/j.amjmed.2008.09.041 19272486

[15] Peterli R Wölnerhanssen BK Peters T Vetter D Kröll D Borbély Y. Effect of laparoscopic sleeve gastrectomy vs laparoscopic roux-en-y gastric bypass on weight loss in patients with morbid obesity the SM-BOSS randomized clinical trial. JAMA (2018) 319:255–65. 10.1001/jama.2017.20897 29340679 PMC5833546

[16] Elahmedi MO Alqahtani AR. evidence base for multidisciplinary care of pediatric/adolescent bariatric surgery patients. Curr Obes Rep. (2017) 6:266–77. 10.1007/s13679-017-0278-3 28755177

[17] Mandl KD Weber GM Natter M Mandel J Adams WG. Scalable collaborative infrastructure for a learning healthcare system (SCILHS): architecture. J Am Med Informat Assoc. (2014) 21:615–20. 10.1136/amiajnl-2014-002727 24821734 PMC4078286

[18] Childerhose JE Alsamawi A Mehta T Smith JE Woolford S Tarini BA. Adolescent bariatric surgery: a systematic review of recommendation documents. Surg Obes Relat Dis. (2017) 13:1768–79. 10.1016/j.soard.2017.08.008 28958402

[19] Inge TH Boyce TW Lee M Kollar L Jenkins TM Brandt ML. Access to care for adolescents seeking weight loss surgery. Obesity (2014) 22:2593–7. 10.1002/oby.20898 25234923

[20] Inge TH Courcoulas AP Jenkins TM Michalsky MP Helmrath MA Brandt ML. weight loss and health status 3 years after bariatric surgery in adolescents. N Eng J Med. (2016) 374:113–23. 10.1056/NEJMoa1506699 26544725 PMC4810437

